# A single-center, observational, retrospective, case control study of rituximab for the treatment of interstitial pneumonia associated with autoimmune features

**DOI:** 10.3389/fphar.2026.1795403

**Published:** 2026-04-07

**Authors:** Tegveer Sandhu, Lea Meir, Nicole Ng, Lila Klein, Maria Padilla, Ioannis Tassiulas

**Affiliations:** 1 Division of Rheumatology, Icahn School of Medicine at Mount Sinai, New York, NY, United States; 2 Division of Pulmonary, Critical Care and Sleep Medicine, Icahn School of Medicine at Mount Sinai, New York, NY, United States

**Keywords:** autoimmune lung disease, CTD-ILD, ILD, interstitial pneumonia with autoimmune featured (IPAF), rituximab

## Abstract

**Background:**

Patients with interstitial lung disease (ILD) and features of autoimmunity who do not meet the classification criteria for a specific autoimmune rheumatic disease are diagnosed with interstitial pneumonia with autoimmune features (IPAF). The treatment approach to ILD in this setting remains undefined. We conducted an observational retrospective study to examine the use of rituximab in IPAF.

**Methods:**

Patients from the Mount Sinai Respiratory Institute Interstitial Lung Disease Registry were included if they met the 2015 classification criteria for IPAF and were treated with at least one dose of rituximab. Clinical improvement was defined as improvement in four domains after the use of rituximab including pulmonary function tests, CT chest findings, need for respiratory related hospitalization and survival.

**Results:**

Of the 791 patients in the registry, 14 patients met the criteria for IPAF and received at least one dose of rituximab. Nineteen patients with IPAF were identified to serve as the control group. More patients in the rituximab group received immunosuppressive medications. The percentage of patients with improved, stable, or worsened pulmonary function tests was similar in both groups. Frequency of oxygen use, incidence of infection, respiratory related admissions and overall mortality was similar in both groups.

**Conclusion:**

The majority of patients with IPAF receiving rituximab showed improvement or stability in their pulmonary function. Although both groups had similar outcomes, more patients in the rituximab group were on baseline immunosuppressive therapy suggesting refractory ILD. We suggest rituximab is a treatment option for patients with moderate to severe IPAF who progress despite standard therapy.

## Introduction

Patients with interstitial lung disease (ILD) and clinical features of autoimmunity who do not meet classification criteria for a specific autoimmune rheumatic disease, such as scleroderma or idiopathic inflammatory myopathy (IIM), are diagnosed with interstitial pneumonia with autoimmune features (IPAF). However, data on the treatment of IPAF are limited to observational studies. Most notably, these studies report the use of corticosteroids and disease-modifying antirheumatic drugs (DMARDs), including mycophenolate mofetil, azathioprine, and tacrolimus. Reported outcomes are heterogeneous and range from relative stability to progressive decline leading to mortality (Joerns et al., 2021; Jiwrajka et al., 2022). To date, there are no data in the current literature on the use of biotherapy in this population, with the exception of a single study (D’Silva et al., 2021). Given that biotherapy has shown benefit in patients with refractory rheumatic disorder–associated ILD, it is therefore reasonable to hypothesize that patients with IPAF may also benefit from such treatment.

Rituximab (RTX), a chimeric monoclonal antibody targeting the CD20 receptor selectively expressed on B lymphocytes, is considered a valuable therapeutic option in ILD associated with rheumatic disorders. Indeed, rituximab has demonstrated benefit in ILD associated with scleroderma, rheumatoid arthritis, IIM, systemic lupus erythematosus (SLE), and Sjögren’s syndrome, although the strongest evidence exists for scleroderma- and IIM-associated ILD (Vacchi et al., 2021). In this context, we conducted a retrospective, comparative study to examine the use of rituximab in patients with IPAF.

## Methods

### Selection of study subjects

The study patients were identified from the Mount Sinai Respiratory Institute Interstitial Lung Disease Registry patients. The registry contains patients diagnosed with ILD based on either HRCT or lung biopsy findings. This study looked at patients enrolled in the registry between 11/26/2014 and 05/10/2022.

Patients were classified as having IPAF if they met the European Respiratory Society/American Thoracic Society research statement criteria (Fischer et al., 2015). Patients were required to undergo chest CT or have histopathological evidence of ILD that was not attributable to another rheumatological cause. The patients also fulfilled the established criteria for at least 2 of the 3 IPAF domains: clinical, serologic, and morphologic. Patients who met the criteria for another autoimmune disorder such as rheumatoid arthritis, scleroderma, IIM, systemic lupus erythematosus, Sjogren’s syndrome, or mixed connective tissue disease were excluded (Lundberg et al., 2017; Shiboski et al., 2017; Van Den Hoogen et al., 2013; Petri et al., 2012; Kay and Upchurch, 2012). Patients who received at least one cycle comprising two infusions of rituximab were included in the rituximab group.

### Data collection

For each patient, a manual chart review was performed. Specifically, the data collected included age, sex, ethnicity, weight, and medical comorbid illnesses, including hypertension (HTN), diabetes mellitus (DM), chronic kidney disease (CKD), obstructive sleep apnea (OSA), chronic obstructive pulmonary disease (COPD), asthma, pulmonary hypertension (PHTN), coronary artery disease (CAD), peripheral vascular disease (PVD), solid tumors, lymphoma, and leukemia. In addition, data was collected on whether patients had clinical features of IPAF, including the presence of Raynaud’s phenomenon, digital fissures (mechanic’s hands), digital edema, digital ulcerations, Gottron’s papules, inflammatory arthritis or polyarticular morning joint stiffness lasting ≥60 min, and palmar telangiectasia. Furthermore, laboratory features of IPAF were recorded, including the presence of antinuclear antibodies (ANA) at a titer ≥1:320, rheumatoid factor ≥2× the upper limit of normal, anti–cyclic citrullinated peptide (anti-CCP), anti–double-stranded DNA (anti-dsDNA), anti-Ro (SS-A), anti-La (SS-B), anti–ribonucleoprotein, anti-Smith, anti-topoisomerase I (Scl-70), anti–tRNA synthetase antibodies (Jo-1, PL-7, PL-12; others include EJ, OJ, KS, Zo, and YRS), anti–PM-Scl, and anti–MDA-5.

The pattern of ILD on imaging was documented and included nonspecific interstitial pneumonia (NSIP), organizing pneumonia (OP), NSIP with OP overlap, and lymphocytic interstitial pneumonia (LIP). When available, histopathologic patterns of ILD were also recorded and included NSIP, OP, NSIP with OP overlap, LIP, interstitial lymphoid aggregates with germinal centers, and diffuse lymphoplasmacytic infiltration, with or without lymphoid follicles. Finally, the use of immunosuppressive medications including rituximab, mycophenolate, tacrolimus, azathioprine, mercaptopurine, intravenous immunoglobulin (IVIG), and cyclophosphamide as well as the use of anti-fibrotic medications, was recorded.

### Respiratory parameters

For all patients who met the criteria for IPAF, baseline and the most recent available pulmonary function tests (PFTs) were recorded. Specifically, the PFT variables included percent predicted forced vital capacity (FVC) and percent predicted diffusing capacity of the lung for carbon monoxide (DLCO). Changes in lung function were assessed by calculating delta FVC (percent predicted) and delta DLCO (percent predicted), which were derived by subtracting the most recent values from the baseline percent predicted FVC and DLCO, respectively. For patients who received rituximab in addition to standard therapy, an additional set of PFTs obtained within 12 months before and after rituximab infusions was recorded. Similarly, for these patients, chest CT scans performed within 12 months before and after rituximab treatment were compared to determine whether findings improved, remained stable, or worsened. In addition, data on the need for supplemental oxygen, documented infections, and noninfectious respiratory-related hospital admissions were collected.

### Statistical analysis

Statistical analyses were performed using IBM SPSS Statistics version 27 (IBM Corp., Armonk, 1989, 2019). Dichotomous variables were reported as raw values and percentages and were compared using the χ2 test. In cases where the 2 × 2 matrices contained cells with expected values of less than 5, Fisher’s exact test was used. The Shapiro–Wilk test was used to assess the normality of continuous variables. For normally distributed variables, an independent sample *t*-test was used to check for statistical significance. For continuous variables that were not normally distributed, we used the Mann–Whitney *U* test to check for statistical significance. The univariate analysis included rituximab, mycophenolate, tacrolimus, glucocorticoids, IVIG, and cyclophosphamide as independent variables. Respiratory outcomes (need for supplemental oxygen, incidence of infections, mortality, and respiratory-related admissions), delta FVC, and delta DLCO were the dependent variables in the univariate analysis. Independent variables with a p-value <0.1 were included in the multiple regression analysis model.

## Results

### Baseline demographics and comorbid conditions

**FIGURE 1:**
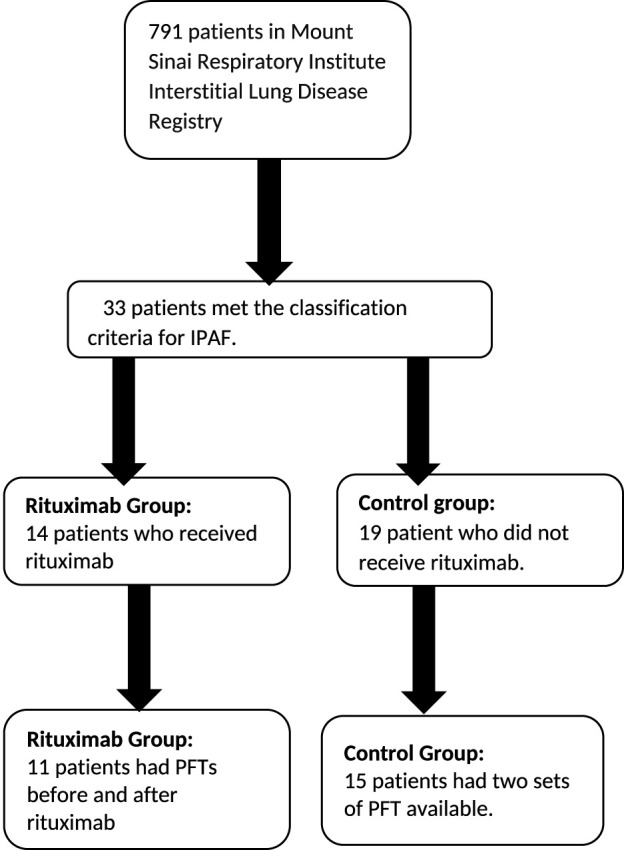
Selection process for the patients in the rituximab and the control group.

As shown in Figure 1, of 791 patients in the Mount Sinai Respiratory Institute Interstitial Lung Disease Registry, fourteen patients met the criteria for IPAF and received rituximab in addition to the standard of care therapy. All but one patient received at least one cycle of rituximab comprising two doses. One of the 14 patients received only one dose and was included in the rituximab group. Nineteen patients who met the criteria for IPAF and received standard care therapy but not rituximab served as the control group (Figure 1). There were no differences in mean age, sex distribution, racial distribution, or comorbid illnesses among the patients (Table 1).

**TABLE 1:** Baseline demographics.

Characteristic	IPAF with rituximab (N = 14)	IPAF without rituximab (N = 19)	P value
Age (mean +SD)	61.6 + 17.5	71.3 + 8.1	0.85
Male	6 (42.9)	9 (47.4)	0.5
BMI (median)	26.2	25	0.5
White	10 (71.4)	13 (68.4)	0.4
African American	2 (14.3)	3 (15.8)	0.4
Other	2 (14.3)	3 (15.8)	0.5
OSA	0	2 (10.5)	0.3
COPD	0	1 (5.3)	0.5
Asthma	1 (7.1)	0	0.4
Pulmonary HTN	1 (7.1)	3 (15.8)	0.4
CAD	0	1 (5.3)	0.5
HTN	2 (14.3)	3 (15.8)	0.6
DM	1 (7.1)	0	0.4
Solid tumor	1 (7.1)	1 (5.3)	0.6
Lymphoma/leukemia	0	1 (5.3)	0.5

### Clinical characteristics

Patients were classified as having IPAF if they met the European Respiratory Society/American Thoracic Society research statement criteria. Table 2 presents patients in both the control and rituximab groups, along with the clinical, serologic, and/or morphologic features observed, as well as the use of immunosuppressive and antifibrotic medications. Overall, no significant differences were observed between groups, except that the proportion of patients with positive anti–tRNA synthetase antibodies was higher in the rituximab group compared with the control group. In addition, more patients in the rituximab group received intravenous immunoglobulin (IVIG). Similarly, the number of patients receiving mycophenolate mofetil was higher in the rituximab group, with a p-value of 0.05.

**TABLE 2:** Clinical characteristics.

Characteristic	IPAF with rituximab (N = 14)	IPAF without rituximab (N = 19)	P value
Clinical criteria for IPAF			
Digital ulcerations	1 (7.1)	0	0.4
Inflammatory arthritis	2 (14.3)	2 (10.5)	0.5
Raynaud’s phenomenon	4 (28.6)	3 (15.8)	0.3
Gottron’s papules	2 (14.3)	2 (10.5)	0.5
Laboratory criteria for IPAF			
ANA positivity	6 (42.9)	7 (36.8)	0.5
ANA titre ≥1:320	3 (21.4)	5 (26.3)	0.5
Anti-centromere	2 (14.3)	0	0.172
RF	0	2 (5.3)	0.496
Anti-CCP positivity	1 (7.1)	3 (15.8)	0.6
Anti-DsDNA	1 (7.1)	1 (5.3)	1
Anti-SSA	4 (28.6)	6 (31.6)	0.5
Anti-SSB	0	1 (5.3)	0.5
Anti-RNP	1 (7.1)	2 (10.5)	0.6
Anti-Scl70	0	1 (5.3)	0.5
Anti-tRNA-synthetase	7 (50)	2 (10.5)	0.017
Anti-PM/Scl	0	1 (5.3)	0.4
Anti-MDA 5	0	1 (5.3)	0.5
Morphologic criteria for IPAF			
NSIP	10 (71.4)	15 (78.5)	0.46
OP	3 (21.4)	0	0.06
NSIP with OP overlap	2 (14.3)	0	0.17
LIP	1 (7.1)	0	0.4
UIP	1 (7.1)	1 (5.3)	0.6
Biopsy	5 (35.6)	7 (36.9)	0.6
NSIP on biopsy	1 (7.1)	1 (5.3)	0.6
OP on biopsy	4 (28.6)	2 (10.5)	0.19
NSIP with OP overlap on biopsy	1 (7.1)	0	0.4
UIP on biopsy	1 (7.1)	1 (5.3)	0.6
Interstitial lymphoid aggregates with germinal centers	1 (7.1)	1 (5.3)	0.6
Diffuse lymphoplasmacytic infiltration	0	1 (5.3)	0.5
Unexplained pericardial thickening or effusion	0	1 (5.3)	0.5
Unexplained pleural thickening or effusion	1 (7.1)	0	0.4
Unexplained intrinsic airway disease (by PFTs, imaging or pathology)	1 (7.1)	1 (5.3)	0.6
Immunosuppressive medications and antifibrotic medication use			
Steroids	11 (78.6)	17 (89.5)	0.3
Mycophenolate	12 (85.7)	7 (36.8)	0.05
Azathioprine	3 (21.4)	0	0.067
Tacrolimus	3 (21.4)	0	0.067
Cyclophosphamide	1 (7.1)	0	0.6
IVIG	4 (28.6)	0	0.024
Pirfenidone	1 (7.1)	1 (5.3)	0.6

#### Respiratory outcomes

**FIGURE 2:**
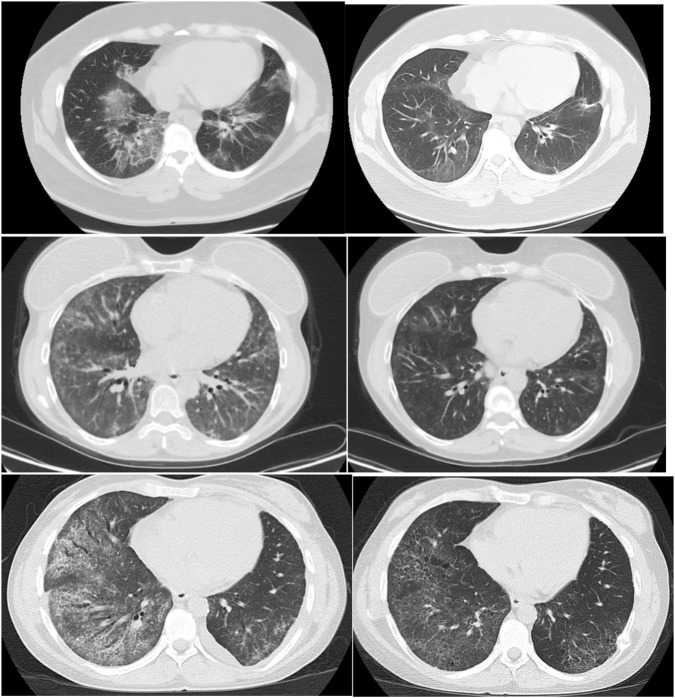
CT scan findings before (left) and after (right) rituximab therapy showing improvement in three of the nine patients who had repeat CT chests within 12 months of rituximab therapy.

Table 3 shows respiratory outcomes in the rituximab and control groups. Eleven patients in the rituximab group had PFTs available within a time frame of 12 months before and after receiving rituximab therapy, whereas fifteen patients in the control group had two sets of PFTs available. Notably, the percentage of patients with improved, stable, or worsened FVC and DLCO was similar between the two groups. Consistent with this finding, there were no differences in baseline, most recent, or delta FVC and DLCO between groups. Nine patients had repeat CT scans of the chest before and after rituximab therapy; CT scans in all but one patient were performed within a time frame of 1 year before and 1 year after treatment. Among these patients, three showed improvement in CT chest findings, four demonstrated stability, and two had worsening of CT chest findings after rituximab therapy. Figure 2 shows repeated chest CT scans of the three patients who demonstrated improvement in their ILD. Moreover, the frequency of oxygen use was similar in both groups. The incidence of infection and respiratory-related admissions was also similar, and there was no difference in mortality between the two groups.

**TABLE 3:** Respiratory outcomes.

Outcome	Rituximab (n = 14)	Control group (n = 19)	P value
Need for supplemental oxygen	5 (35.7)	9 (47.4)	0.3
Incidence of infections	6 (42.9)	5 (26.3)	0.2
Respiratory related admissions	2 (14.3)	5 (26.3)	0.3
Mortality	1 (7.1)	1 (5.3)	0.5
Pulmonary function tests			

​	Rituximab (n = 11)	Control group (n = 15)	P value
Percent predicted FVC improved	5 (45.4)	4 (26.6)	0.4
Percent predicted FVC remained stable	4 (36.3)	7 (46.6)	0.5
Percent predicted FVC worsened	2 (18.1)	4 (26.6)	0.7
Baseline percent predicted FVC	72.4 + 18.2	69.6 + 18.7	0.68
Most recent percent predicted FVC	69.6 ± 15.7	73.4 ± 28.5	0.68
Percent predicted FVC delta	−2.57 ± 24	2.22 ± 14.5	0.8
Median years between the PFTs	3.1	3.3	0.5

​	Rituximab (n = 10)	Control group (n = 15)	P value
Percent predicted DLCO improved	3 (30)	4 (26.6)	0.63
Percent predicted DLCO stable	5 (50)	8 (53.3)	0.4
Percent predicted DLCO worsened	2 (20)	3 (20)	0.6
Baseline percent predicted DLCO	51 + 17.3	54.6 ± 19.4	0.9
Most recent percent predicted DLCO	53.7 + 19.3	52.7 ± 25.9	0.7
Percent predicted DLCO delta	2.6 ± 13.2	−1.87 + 16.9	0.6

### PFTs trend in the rituximab group

**FIGURE 3:**
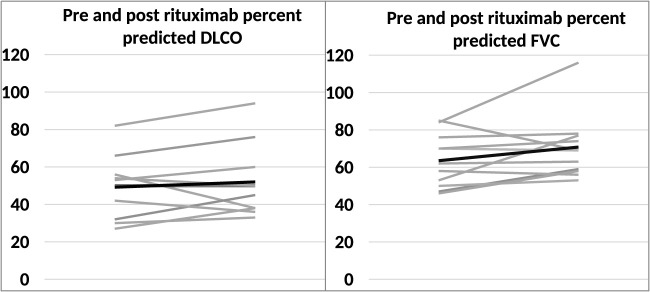
Line diagram showing the trend of the percent predicted DLCO and FVC before and after rituximab. Mean percent predicted DLCO and FVC values are represented by the solid black line.

The trends in percent predicted DLCO and FVC are shown in Figure 3. The mean percent predicted FVC and DLCO before rituximab infusion were 63.6 ± 13.6 and 51.0 ± 17.3, respectively. After treatment, the mean percent predicted FVC and DLCO values were 70.8 ± 16.6 and 53.7 ± 19.3, respectively. Although both percent predicted FVC and DLCO demonstrated an upward trend after rituximab therapy, these changes were not statistically significant.

### Management of baseline immunosuppressant medications in rituximab group

Preexisting immunosuppressive medications were continued after the initiation of rituximab in 10 of the 14 patients who received it. Immunosuppressive medications were discontinued in three patients. One patient was started on rituximab first, and an additional immunosuppressive agent was administered after rituximab initiation.

### Multiple regression analysis

We performed an initial univariate analysis that screened several independent variables, including rituximab, mycophenolate, tacrolimus, glucocorticoids, IVIG, and cyclophosphamide. Variables demonstrating a trend toward significance (p < 0.1) were included in the final multiple linear regression models.

Oxygen use: Age was found to be a negative predictor (B = −0.016, p = 0.014). Patients who were administered mycophenolate showed significantly higher oxygen use (B = 0.467, p = 0.015). Tacrolimus use was not significant in the multivariate analysis (B = 0.579, p = 0.053).

Mycophenolate use was a significant independent predictor of infection risk in the model (B = 0.458, p = 0.008). Patients on mycophenolate had a significantly higher infection risk than those who did not, with the constant use of azathioprine. Azathioprine (B = 0.124, p = 0.650) and age (B = 0.001, p = 0.840) were not significant predictors of infection in the multivariate regression analysis.

## Discussion

Studies examining the treatment of interstitial pneumonia with autoimmune features are very limited and are primarily retrospective observational in nature. A study conducted by Karampeli et al. evaluated the response to immunosuppressive treatment in 39 IPAF patients and reported stabilization or improvement in 79.5% of patients, despite fewer immunosuppressive medications being used compared with our study. In their cohort, 7.9% of patients received mycophenolate mofetil, 21% received azathioprine, 7.9% received hydroxychloroquine in combination with azathioprine, 23.7% received methotrexate, and 7.9% received cyclophosphamide (Karampeli et al., 2020). Notably, the favorable outcomes observed despite fewer immunosuppressive agents may be explained by higher baseline pre-treatment PFTs, with a mean % predicted FVC of 79%.

Another study conducted by Joerns et al. evaluated 63 IPAF patients with substantially lower baseline PFTs (mean FVC 59.04% ± 14.53% and mean DLCO 41.87% ± 16.67%). In this cohort, 84% of patients were treated with immunosuppressive medications, including prednisone with mycophenolate mofetil (69%), prednisone with azathioprine (20.6%), and prednisone combined with mycophenolate mofetil and azathioprine (6.35%). Importantly, a decrease in the rate of ILD progression was observed in 69% of patients treated with prednisone and mycophenolate mofetil (76.47% vs. 41.67%, p = 0.018), which is consistent with the established therapeutic benefit of MMF in CTD-ILD (Jiwrajka et al., 2022). DaSilva et al. evaluated 50 IPAF patients across two medical centers, including 36 patients at Mass General Brigham (MGB) and 14 patients at the University of Chicago Medicine (UCM) (D’Silva et al., 2021). Patients at UCM had more severe ILD, with a mean % predicted FVC of 49% and % predicted DLCO of 35%, compared with MGB patients (mean % predicted FVC of 66% and % predicted DLCO of 45%). Patients in the UCM group continued to progress despite a substantial proportion receiving MMF (64%), AZA (43%), and tacrolimus (64%), and therefore required initiation of rituximab, with discontinuation of prior immunosuppressive therapy in the majority of cases. Following rituximab initiation, approximately 71% of patients achieved improvement or stability.

In contrast, the clinical course of patients in the MGB group differed, with fewer patients requiring MMF (11%), AZA (3%), leflunomide (3%), or cyclophosphamide (9%). Except for a higher percentage of patients continuing MMF (31%), all other immunosuppressive therapies were discontinued after rituximab initiation, with 83% of patients remaining stable or demonstrating improvement. Taken together, these findings highlight the efficacy of rituximab both as an initial therapy in moderate ILD and as an alternative therapy in severe ILD refractory to first-line immunosuppressive agents. Collectively, these studies demonstrate the benefit of standard DMARD therapy, particularly in patients with milder ILD. However, as suggested by the study conducted by DaSilva et al., this benefit may be limited, and additional biotherapy such as rituximab may be required to halt disease progression in certain patients. Given the extremely limited data on biotherapy in IPAF, inferences must also be drawn from studies of CTD-ILD, most commonly associated with scleroderma and idiopathic inflammatory myopathies.

Initial therapy for CTD-ILD typically includes glucocorticoids and immunosuppressive agents such as mycophenolate mofetil and azathioprine, with rituximab reserved for refractory disease (Antin-Ozerkis and Hinchcliff, 2019). An open-label multicenter study conducted by Daoussis et al. followed 51 patients with SSc-ILD for a median of 4 years and demonstrated an increase in FVC and DLCO from baseline (Daoussis et al., 2017). Thirty-three patients received rituximab either as monotherapy or as an add-on to ongoing treatment, while 18 patients continued prior therapy. Patients treated with rituximab showed a significant increase in FVC at 24 months. Although there was also a trend toward higher FVC and DLCO compared with the control group, this difference was not statistically significant.

In our study, the rituximab group demonstrated a numeric decline between baseline and the most recent FVC over a median interval of 3.1 years; however, this difference was not statistically significant. In contrast, PFTs obtained within 1 year of rituximab infusion demonstrated a trend toward improvement in both FVC and DLCO. Daoussis et al. reported a similar observation in six patients whose PFTs declined after temporary discontinuation of rituximab and improved upon resumption, suggesting that sustained treatment may be required for durable benefit.

It is important to note that not all studies of scleroderma-associated ILD demonstrate significant improvement. A prospective study of 643 patients with ILD-SSc from the EUSTAR cohort reported stability in both rituximab-treated and untreated groups (Elhai et al., 2019). Patients in the rituximab group had lower baseline FVC values, indicating more severe disease, suggesting that stability itself may represent a favorable outcome in advanced ILD. Our study similarly demonstrated comparable proportions of patients with improved, stable, or worsened PFTs in both the rituximab and control groups. Baseline demographics, including age, sex, racial distribution, comorbid illnesses, and clinical and radiologic features, were comparable between groups, as were baseline PFTs, suggesting similar disease severity at study entry. Nevertheless, more patients in the rituximab group had previously received other DMARDs, including MMF, tacrolimus, and IVIG, potentially reflecting a more aggressive disease course.

Given that rituximab is typically reserved for patients who have failed prior therapies, it is reasonable to infer that this group represents a cohort with more progressive disease, which aligns with existing data demonstrating benefit in severe CTD-ILD. Our study also demonstrated improvement in three of nine patients who underwent repeat chest CT imaging before and after rituximab therapy, with four patients showing stability and two demonstrating worsening. Notably, the control group did not undergo follow-up CT imaging. Importantly, most patients were already receiving immunosuppressive therapy prior to rituximab initiation, suggesting refractory ILD; therefore, it is reasonable to attribute the observed radiographic improvement to rituximab therapy. A retrospective study by Jiwrajka et al. evaluated 456 patients from a single-center ILD cohort and identified 60 patients with IPAF (Van Den Hoogen et al., 2013). Male sex was independently associated with worse transplant-free survival, whereas no clinical, serologic, or radiologic features were associated with prognosis.

At present, there are no established predictors of survival in IPAF. In our study, no differences in demographics, clinical features, or radiologic characteristics were observed between groups, although patients in rituximab group had a higher prevalence of anti–tRNA synthetase antibodies. Jiwrajka et al. reported that CTD-ILD patients with anti-synthetase and anti-MDA5 antibodies were more likely to present with ILD prior to or concurrent with CTD diagnosis, although the relationship with disease severity remains unclear (Sircar et al., 2018). Similarly, studies evaluating predictors of response to immunosuppressive therapy in ILD have failed to identify consistent associations. Joerns et al. conducted a single-center observational study of 63 IPAF patients and found no statistically significant relationship between baseline clinical, serologic, or morphologic features and treatment response (Shiboski et al., 2017). Interestingly, a trend toward greater disease progression was observed in patients negative for anti–tRNA synthetase antibodies. This contrasts with our findings, in which the rituximab group had a higher prevalence of anti–tRNA synthetase antibodies and required more intensive immunosuppressive therapy, suggesting more severe disease.

Several limitations of our study should be acknowledged. First, this was a retrospective observational study rather than a randomized clinical trial, resulting in differences between groups in age and the use of immunosuppressive medications. Although only the use of IVIG showed statistical significance, these differences may have clinical implications. Inherent differences, such as the severity of illness and disease progression, could have influenced the decision to use rituximab. Second, due to the observational nature of the study, the IPAF criteria were applied retrospectively, and certain laboratory results related to the immunologic criteria may not have been obtained, while clinical criteria may not have been documented. Additionally, the PFTs in our study were not obtained within a prespecified time range, potentially confounding an accurate comparison between the two groups. Finally, the sample size in our study may have been too small to detect statistically significant differences between the groups.

## Conclusion

In conclusion, our study shows improvement or stability in pulmonary function tests for the majority of patients with IPAF receiving rituximab. To our knowledge, this is the first study comparing the use of rituximab with an active control group. Although both groups had similar baseline pulmonary function tests and clinical outcomes, a greater number of patients in the rituximab group were on baseline immunosuppressive medications, indicating refractory ILD. We suggest that rituximab is a valuable treatment option for patients with moderate to severe IPAF who progress despite standard DMARD therapy. Notably, there was no increase in adverse events associated with rituximab use. Further prospective studies and randomized controlled trials are needed to determine the effectiveness of biotherapy, such as rituximab, in IPAF and to identify which clinical, serological, and radiological phenotypes of IPAF might predict response to therapy.

## Data Availability

The datasets presented in this article are not readily available because of patient confidentiality. Requests for accessing the datasets should be directed to TS, tegveer.sandhu@utoronto.ca.

## Author’s note

Data already published in ACR 2023 conference: Sandhu T, Meir L, Ng N, Klein L, Zatakia J, Padilla M, Tassiulas I. A Single-center, Observational, Retrospective, Case Control Study of Rituximab for the Treatment of Interstitial Pneumonia Associated with Autoimmune Features [abstract]. Arthritis Rheumatol. 2023; 75 (suppl 9). https://acrabstracts.org/abstract/a-single-center-observational-retrospective-case-control-study-of-rituximab-for-the-treatment-of-interstitial-pneumonia-associated-with-autoimmune-features/. Accessed 21 September 2023.
